# Immune Modulating Topical S100A8/A9 Inhibits Growth of *Pseudomonas aeruginosa* and Mitigates Biofilm Infection in Chronic Wounds

**DOI:** 10.3390/ijms18071359

**Published:** 2017-06-26

**Authors:** Hannah Trøstrup, Christian Johann Lerche, Lars Christophersen, Peter Østrup Jensen, Niels Høiby, Claus Moser

**Affiliations:** 1 Department of Clinical Microbiology, Copenhagen University Hospital, Rigshospitalet, 2100 Copenhagen, Denmark; cjl@dadlnet.dk (C.J.L.); lars.christophersen@regionh.dk (L.C.); peter.oestrup.jensen@regionh.dk (P.Ø.J.); hoibyniels@gmail.com (N.H.); moser@dadlnet.dk (C.M.); 2 Institute for Immunology and Microbiology, University of Copenhagen, 2100 Copenhagen, Denmark

**Keywords:** biofilm infection, chronic wounds, host defense, *Pseudomonas aeruginosa*, S100A8/A9

## Abstract

*Pseudomonas aeruginosa* biofilm maintains and perturbs local host defense, hindering timely wound healing. Previously, we showed that *P. aeruginosa* suppressed S100A8/A9 of the murine innate host defense. We assessed the potential antimicrobial effect of S100A8/A9 on biofilm-infected wounds in a murine model and *P. aeruginosa* growth in vitro. Seventy-six mice, inflicted with a full-thickness burn wound were challenged subcutaneously (s.c.) by 10^6^ colony-forming units (CFUs) of *P. aeruginosa* biofilm. Mice were subsequently randomized into two treatment groups, one group receiving recombinant murine S100A8/A9 and a group of vehicle controls (phosphate-buffered saline, PBS) all treated with s.c. injections daily for up to five days. Wounds were analyzed for quantitative bacteriology and contents of key inflammatory markers. Count of blood polymorphonuclear leukocytes was included. S100A8/A9-treatment ameliorated wound infection, as evaluated by quantitative bacteriology (*p* ≤ 0.05). In vitro, growth of *P. aeruginosa* was inhibited dose-dependently by S100A8/A9 in concentrations from 5 to 40 μg/mL, as determined by optical density-measurement (OD-measurement) and quantitative bacteriology. Treatment slightly augmented key inflammatory cytokine Tumor Necrosis Factor-α (TNF-α), but dampened interferon-γ (IFN-γ) levels and blood polymorphonuclear count. In conclusion, topical S100A8/A9 displays remarkable novel immune stimulatory and anti-infective properties in vivo and in vitro. Importantly, treatment by S100A8/A9 provides local infection control. Implications for a role as adjunctive treatment in healing of chronic biofilm-infected wounds are discussed.

## 1. Introduction

Infected chronic wounds are an increasing threat to society and can be fatal for diabetic patients. There is an urgent need for adjunctive therapies taking increasing microbial resistance into consideration. Innate host mechanisms may hold a key to this progress in the understanding of wound chronicity.

A rigorous debridement is necessary for optimal treatment of chronic human wounds. Even though surgical effort has been made in order to optimize the wound bed for healing, failure of split-skin transplantation is often the case, especially when *Pseudomonas aeruginosa* biofilm is present in the wound [1]. *P. aeruginosa* biofilms reside deeply in the wound bed [2] and have an undisputable effect on local host response, as increasingly evidencesd in the last decade [3]. Biofilms are invisible to the naked eye and at present, there is no local marker to guide clinicians. Thus, greater understanding of the clinical impact of biofilms on host response is of importance and may lead to the development of new adjunctive treatment strategies that take sufficient antibiotic therapy as well as the immunomodulatory effect of biofilm infections into consideration [4]. 

S100A8 and A9 are the most abundant cytoplasmic proteins of neutrophils and monocytes [5] and are released upon activation of these phagocytes [6]. They form the physiologically relevant heterodimeric S100A8/A9, where S100A8 is the active component and S100A9 the regulating subunit [7]. S100A8/A9 has wide antifungal and antimicrobial potential [8]. We previously reported reduced S100A8/A9 levels in colonized human chronic wounds [9,10]. Since *P. aeruginosa* is able to degrade cytokines [11], S100A8/A9 may likewise be degraded in vivo in anatomically close proximity to the *P. aeruginosa* biofilm as part of the evasion of the immune system. 

S100A8/A9 may exert its host defense functions locally upon neutrophil death mediated release at sites of tissue infection, thereby controlling growth of *P. aeruginosa* [12]. Animal models may shed light on the complex pathophysiology of chronic wounds and accordingly, possible immunomodulatory and beneficial effects of S100A8/A9 on wound infection were investigated in the present study using a representative animal model. The BALB/c strain of mice is susceptible to *P. aeruginosa* infection and literature describes a potential anti-infective effect of this protein. Thus, we assessed the impact of recombinant S100A8/A9 on the course of infection in BALB/c mice as well as the direct inhibitory effect on S100A8/A9 in vitro. 

## 2. Results

### 2.1. Quantitative Bacteriology of P. aeruginosa Biofilm-Infected Wounds

In order to determine an anti-infective effect of host response S100A8/A9 on chronic *P. aeruginosa* biofilm-infected wounds in vivo, we subjected BALB/c mice to topical intervention by this recombinant protein.

**Figure 1:**
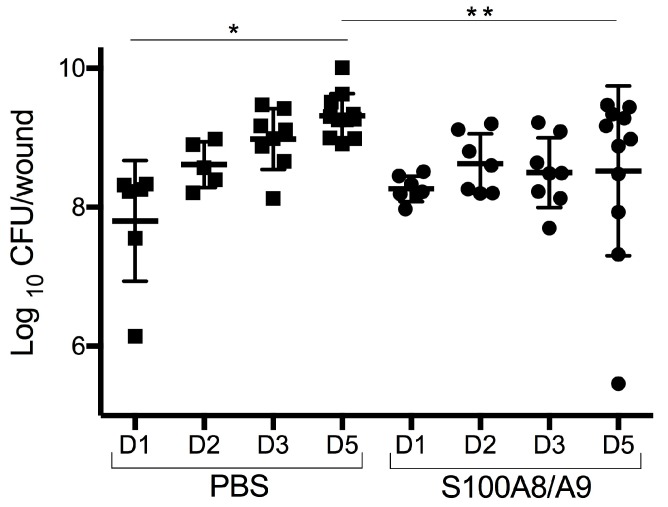
Induction of local wound infection control. Log_10_ colony-forming units (CFUs) of *P. aeruginosa*/wound as expression of time in the intervention group (S100A8/A9) and control group (phosphate-buffered saline, PBS). S100A8/A9-treatment induced infection control. The number of colony-forming units (CFUs) after 5 days of treatment was significantly reduced as compared to the non-treated control group (** *p* < 0.05). Increase in growth over 5 days in the control group only (* *p* < 0.0001). Individual values indicate mean of duplicates with overall mean ± standard deviation. Black squares, PBS-treated; black circles, S100A8/A9-treated.

A significant increase in colony-forming unit (CFU) count from day 1 to day 5 was observed in the control (phosphate-buffered saline, PBS) group (*p* < 0.0001). In contrast, no such increase was seen in the S100A8/A9-treated group. Mean values (log_10_ CFU/wound) for the treated group compared to controls was 8.266 ± 0.183 vs. 7.804 ± 0.866 at day 1. At day 2, it was 8.628 ± 0.429 to 8.618 ± 0.328; day 3, 8.498 ± 0.500 to 8.981 ± 0.437; and day 5, 8.523 ± 1.221 to 9.317 ± 0.316. At the termination of the experiment on day 5, significantly reduced CFUs were found in the S100A8/A9-treated wounds compared to the control group (Figure 1, *p* < 0.05). 

### 2.2. Bacterial Growth Inhibition Assay

**Figure 2:**
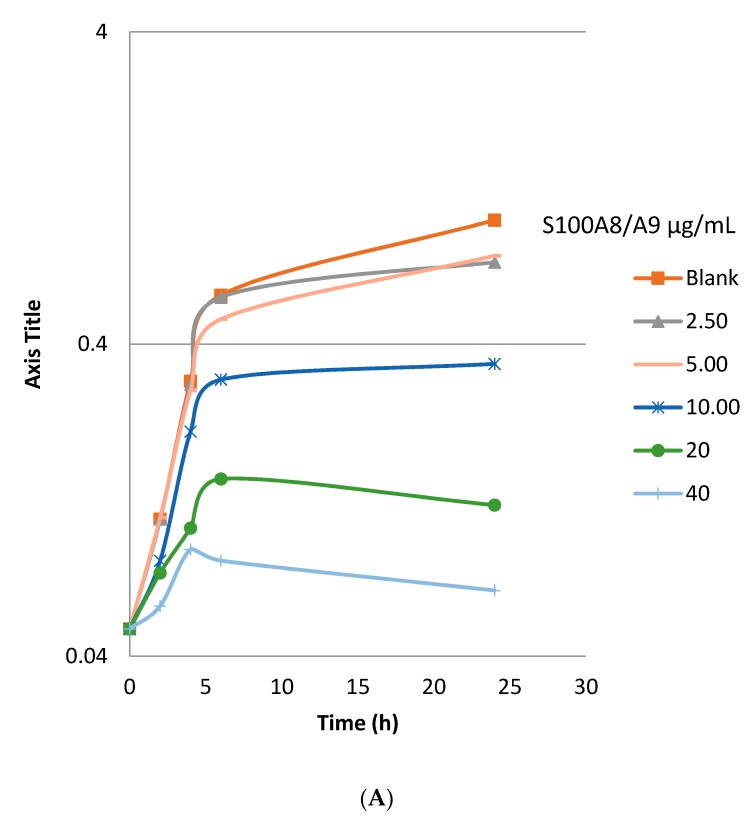

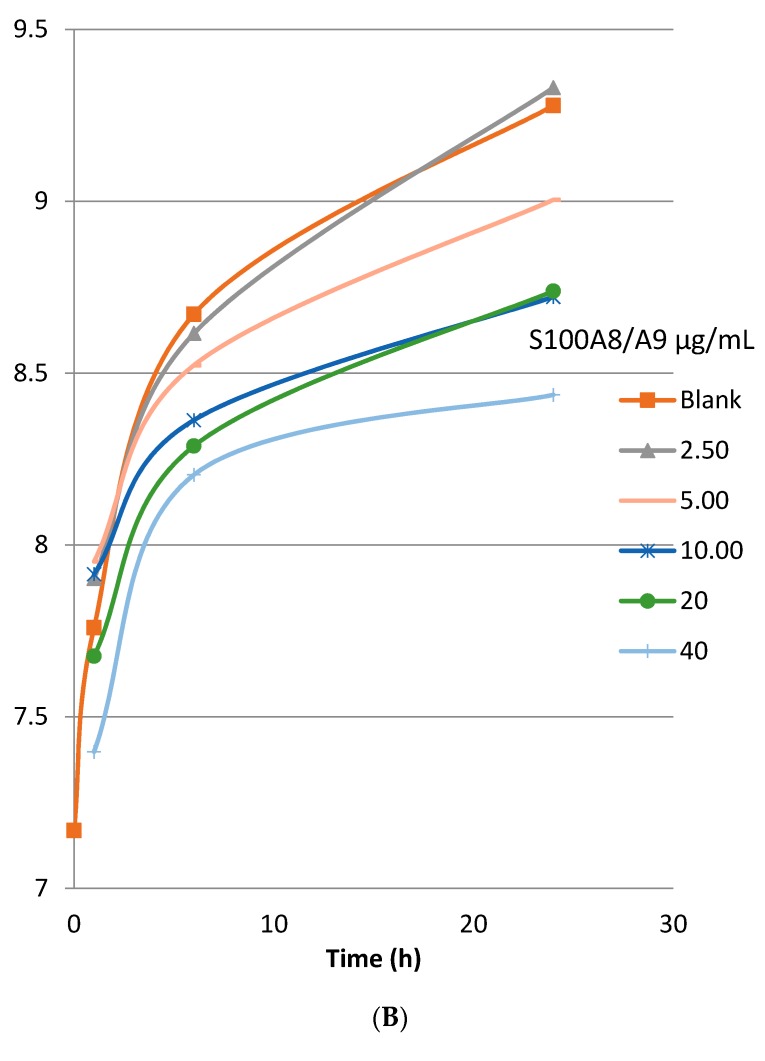
S100A8/A9 inhibits growth of *Pseudomonas aeruginosa*. (**A**) Growth inhibitory effect of S100A8/A9 on PAO1, shown by OD-curve (read at OD_600_ at time points indicated). (**B**) Growth inhibitory effect of S100A8/A9 on PAO1, shown by bacterial CFU count (1 h, 6 h, 24 h, at concentrations 40, 20, 10, 5, 2.5, or 0 μg/mL). Growth inhibitory effect of S100A8/A9 on PAO1, shown by (**A**) OD-curve (read at OD_600_ at time points indicated) and (**B**) bacterial count as log_10_ CFU (1 h, 6 h, 24 h at concentrations 40, 20, 10, 5, 2.5, or 0 μg/mL).

To verify the anti-infective effect of topical S100A8/A9 on infected wounds, we assessed the direct interaction of S100A8/9 and planktonic PAO1 (a strain of *P. aeruginosa*). A growth inhibition assay was performed to determine whether the reduced bacterial levels in the wounds were due to immune modulation or direct inhibition of *P. aeruginosa* growth. The addition of S100A8/A9 reduced bacterial growth of *P. aeruginosa* time- and dose-dependently, starting at 2.50 μg/mL at 24 h of incubation (Figure 2A, optical density (OD) 0.732 versus 0.999 at blank), 5 μg/mL at 6 h (0.481 versus 0.574) and 10, 20, and 40 μg/mL at 2 h (0.081 versus 0.11). In addition, PAO1 was grown in medium for a maximum of 24 h at 37 °C with or without S100A8/A9. CFU counts the following day confirmed the result with a lower CFU/mL at 1 h of incubation with 20 and 40 μg/mL. At 6 h, bacterial growth was inhibited dose-dependently, from 5 to 40 μg/mL (Figure 2B) and a maximal 0.5 log inhibition with 40 μg/mL. At 24 h, approximately 1 log reduction of CFU/mL was observed.

### 2.3. The Impact of S100A8/A9-Treatment on Local Wound Inflammatory Markers

We previously described an aggravated Interleukin-1β (IL-1β) response towards *P. aeruginosa* biofilm in the chronic wound model using BALB/c mice [13]. As S100A8/A9 is an endogenous peptide, we speculated that exogenous application would immunomodulate host defense in the wounds.

**Figure 3:**
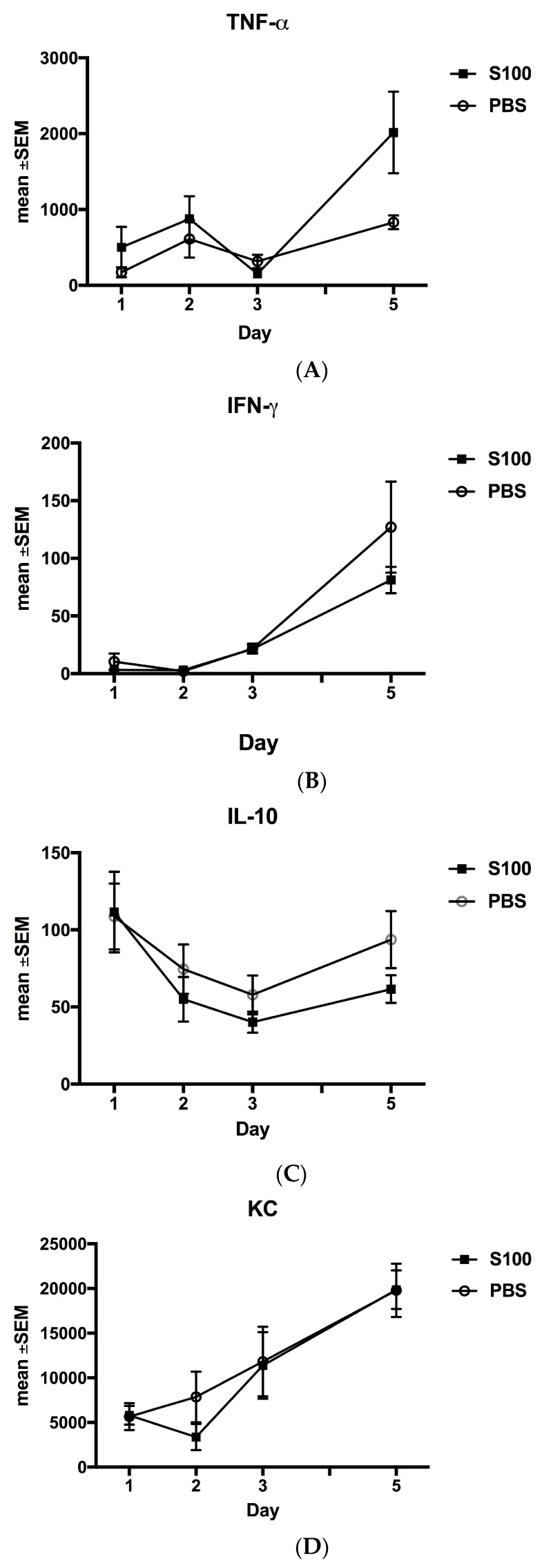

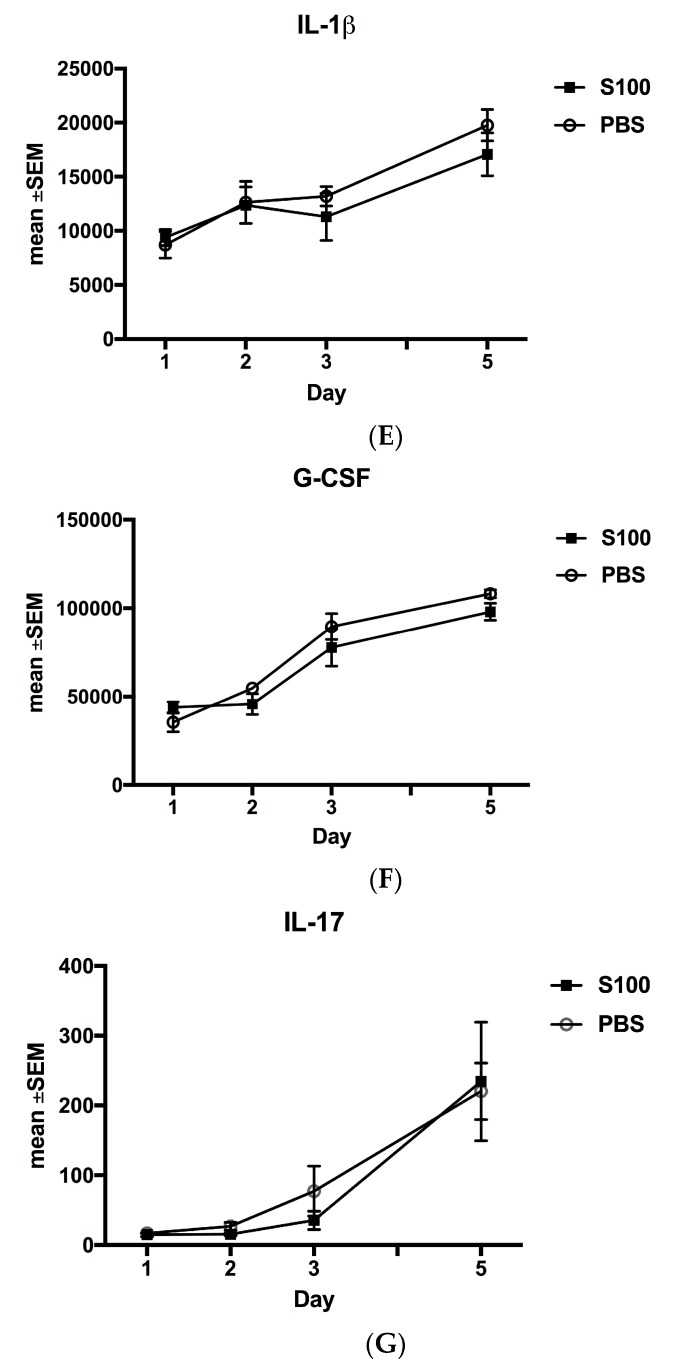
Modulation of proinflammatory cytokines. (**A**) Tumor Necrosis Factor-α (TNF-α) levels were increased in the S100A8/A9 group from day 1 to 5 (*p* < 0.0261) and day 3 to 5 (*p* < 0.001). No such increase was observed in the PBS group; (**B**) Interferon-γ (IFN-γ) levels were increased in the PBS from day 1 to 5 (*p* < 0.0023), 2 to 5 (*p* < 0.0008), and day 3 to 5 (*p* < 0.0054). No increase was observed in the S100A8/A9 group; (**C**) IL-10. No significant changes were observed in either of the two groups, but there was a tendency to an attenuated response in the S100A8/A9 group; (**D**) in the PBS group, Keratinocyte-derived chemokine (KC) increased from day 1 to 5 (*p* < 0.0156). In the S100A8/A9 group, KC increased from day 1 to 5 (*p* < 0.0139) and day 2 to 5 (*p* < 0.0017). (**E**) In both groups, IL-1β increased from day 1 to 5 (*p* < 0.0005 in the PBS group and *p* < 0.0482 in the S100A8/A9 group); (**F**) In both groups, Granulocyte-Colony Stimulating Factor (G-CSF) increased with time for all time points evaluated. (**G**) In both groups, Il-17 increased with time for all time points evaluated. Modulation of inflammatory response by box plots of inflammatory cytokines (**A**–**E**): Levels of proteins are expressed in ρg/mL wound.

S100A8/A9 stimulated Tumor Necrosis Factor-α (TNF-α) from day 1 to 5 (*p* < 0.0261) and 3 to 5 (*p* < 0.001) (Figure 3A). Interferon-γ (IFN-γ) levels (Figure 3B) were discretely attenuated with a significant increase from day 1–5, day 2–5, and day 3–5 only in the control group (*p* < 0.0023, *p* < 0.0008, *p* < 0.0054, respectively). In the S100A8/A9 group, no such increase was seen.

There were no significant immunomodulatory effects of S100A8/A9 on the levels of IL-1β (Figure 3C), IL-10 (Figure 3D), Keratinocyte-derived Chemokine (KC) (Figure 3E), Granulocyte-Colony Stimulating Factor (G-CSF) levels (Figure 3F), or IL-17 levels (Figure 3G).

IL-1β, IL-10, KC, and G-CSF levels were all induced by infection (Figures S2–S5). IL-1β levels were induced in 1 day with treatment or PBS, TNF-α in 5 days with treatment or PBS as compared to background mice (Figures S1 and S2). 

### 2.4. Systemic Polymorphonuclear Count

**Figure 4:**
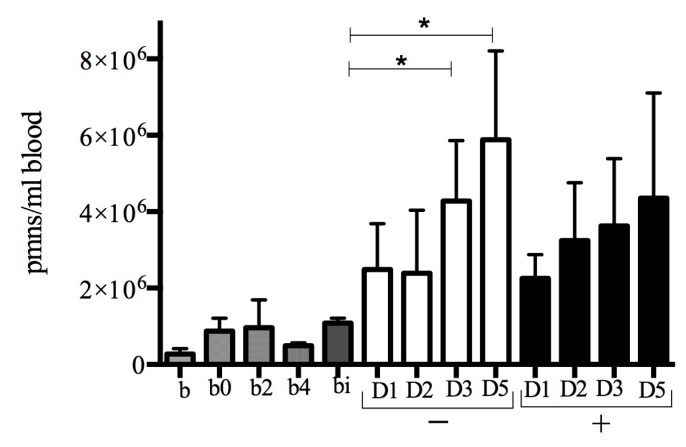
S100A8/A9 dampens systemic polymorphonuclear count. Systemic polymorphonuclear (PMN) count in S100A8/A9 (+) treated group versus PBS (−). Bars are expressed with mean + standard deviation. No significant increase was seen in the S100A8/A9 group. In contrast, in the PBS group, an increase in PMN count was seen from day 1 to 3 (*p* < 0.03) and day 1 to 5 (*p* < 0.003). Abbreviations: b: background (*n* = 6), b0: burned, results obtained immediately after wounding, b2: burned, two days after wounding, b4: burned, 4 days after wounding; bi: burn and infection, results obtained immediately after infection. D1, 2, 3, 5: day 1, 2, 3, 5. *, significant increase.

In the S100A8/A9 group, no significant increase in PMN count was observed from 1 to 5 days of treatment. In contrast, in the control group, an increase in PMN count was seen from 1 to 3 days after infection (*p* < 0.03) and from 1 to 5 days of treatment (*p* < 0.003). Infection induced significantly higher PMN counts in all groups compared to background mice (no wound, no infection, *n* = 6, mean PMNs/mL whole blood ± SD: 2.7 × 10^5^ ± 1.4 × 10^5^). Burn wounding (controls immediately after wound infliction (*n* = 3), and at 2 (*n* = 3) and 4 days (*n* = 3) post wound infliction) and burn wounded mice challenged with infection and sacrificed immediately after infection (*n* = 3) induced higher PMN count than in background mice (Figure 4). Systemic total white blood cell count at 5 days after infection was equal between the groups (Figure S6).

### 2.5. Digital Planimetry

To assess a clinical impact on wound closure, we analyzed wound area and necrotic areas by planimetry.

After 5 days of treatment, S100A8/A9 did not affect the total wound area (mean 244.9 mm^2^ ± 74.11 versus 257.1 ± 60.54 for the control group) or the area of necrosis at day 5 (174.8 mm^2^ ± 52.38 versus 180.1 ± 34.27 for the control group) (Figures S7 and S8).

### 2.6. Epidermal Growth Factor Levels

The key growth factor epidermal growth factor (EGF) was quantified as a pseudomarker for wound closure, with results showing no effect of S100A8/A9 on EGF levels in wounds (Figure S9).

## 3. Discussion

The aim of the present study was to evaluate an antimicrobial effect in vivo and in vitro of S100A8/A9 on *P. aeruginosa*, a clinically important wound pathogen.

Selection of this calcium-binding protein [14] was based on a previous description of reduction of S100A8/A9 in human chronic wounds [9,10].

Degradation of S100A8/A9 in the proteolytic wound environment could explain this reduction. However, the protein displays high protease-resistance, suggesting another route [15]. Regardless of the underlying mechanism behind S100A8/A9 reduction in chronic biofilm-infected wounds, a logical consequence of previous observations was to perform a study of topical S100A8/A9 treatment with dose selection of 1 μg/250 μL. Studies performed by Sroussi et al. showed that a single dose of topically applied ala^42^ S100A8 (25 μL of a 1 μg/mL solution intradermal +10 μL directly to excisional murine wounds) reduced bacterial CFU count in mice subject to restraint stress at day 1 and day 5 post wounding. However, the bacteriology was not further described and biofilm presence, in contrast to the present study, was not considered [16].

The present results show, to our knowledge, a novel inhibitory effect of S100A8/A9 on *P. aeruginosa* in a murine model of biofilm-infected wounds as well as in vitro. Growth inhibition by S100A8/A9 has been described by several groups, in multiple species in vitro including *Borrelia burgdorferi*, *Klebsiella pneumoniae*, *Escherichia coli* [12], *Staphylococcus aureus*, *Candida albicans*, and *Salmonella typhimurium* [8,17,18,19,20]. The addition of zinc in several of the studies reversed the inhibitory effect and accordingly, S100A8/A9 chelation of zinc and manganese [8] may inhibit growth by nutritional immunity. Another possibility could be the disruption of biofilm alginate polymerization by S100A8/A9 bound calcium and a resultant increase in susceptibility of bacteria superficially situated in the biofilm to phagocytosis by PMNs. Another potential method of action was described by Akerström et al. [21], who found a lysing effect of S100A8/A9 on the anaerobic bacterium *Finegoldia magna* (previously known as *Peptostreptococcus magnus*) by interacting with the cell membrane.

Regarding the modulation of inflammatory mediators in the wounds, treatment augmented TNF-α and decreased IFN-γ levels locally. Vogl and colleagues showed that S100A8/A9 amplifies phagocyte activation during lipopolysaccharide-induced sepsis via activation of TLR-4 upstream of TNF-α response [7]. The reason for a potential decrease in IFN-γ is not clear. We assessed the wounds in the early part of inflammation and speculate that at this point IFN-γ acts as an acute inflammatory marker, related to the wound infliction itself. We have previously shown an improved clinical outcome of lung disease in Th1-reacting C3H/HeN mice (higher IFN-γ levels) as compared to Th2-reacting BALB/c mice [22,23]. The adaptive immune response also emerges only at the termination of the present study. Accordingly, the timing of treatment and improved healing outcome and correlations to IFN-γ levels need further investigation.

Previously, we found an early aggravating effect of *P. aeruginosa* biofilm on local IL-1β levels in BALB/c mice. Treatment by S100A8/A9 in the doses chosen in this study did not counteract this observation. 

In regard to the stability and pharmacodynamics of S100A8/A9, we were unable to find reports on the durability of exogenously applied S100A8/A9 in vivo, but further experiments could address this matter. In vitro, S100A8/A9 is extremely protease-resistant [15].

In addition to growth inhibiting properties, S100A8/A9 may also have immunomodulatory leukocyte effects [24]. In a murine model of *P. aeruginosa* keratitis, S100A8/A9 treatment promoted bacterial clearance by increasing production of reactive oxygen species [25]. Interestingly, subunit S100A9 induces neutrophil phagocytosis of *Eschericia coli* [26]. Therefore, an increased phagocytosis caused by appropriate levels of S100A8/A9 might be an additional explanation for the diminished bacterial count in the present study. A role for S100A8/A9 in the oxidative metabolism and functional role of neutrophils should be explored further, as this could explain the reduced CFU count in the S100A8/A9-treated group of mice and may indirectly impact the delayed IL-1β response. Ryckmann et al. showed that S100A8/A9 in the concentration range 10^−12^ to 10^−10^ M acts as a chemotactic factor for neutrophils in vitro. In the same study, equimolar quantities of S100A8 and A9 (0.1–10 μg/mL), dissolved in PBS, attracted neutrophils in an in vivo murine air pouch model [27]. 

In the present study, treatment reduced blood PMN count although this attenuation was not reflected in the PMN mobilizer G-CSF, nor the two chemo-attractants KC and IL-17. Interestingly, no significant differences in levels of cytokines and chemokines were found between treated and control groups while these inflammatory markers increased with time in both groups. We speculate that the dose of S100A8/A9 chosen was insufficient to cause significant differences. Ongoing studies will substantiate relevant and safe doses of S100A8/A9 in this model.

A reduction of circulating PMNs could theoretically be beneficial, as chronic wounds are trapped in the inflammatory state of healing, showing continuous neutrophil extravasation and excessive host responses regarding TNF-α, but not IL-17, which is part of the adaptive immune system. IL-17 suppression by S100A8/A9 could imply that this protein delays initiation of the adaptive immune response. We hypothesize that S100A8/A9 is a marker of activated, phagocytizing leukocytes. The reduced extracellular levels of this protein, observed in our previous studies, may reflect the inhibition of neutrophil function and phagocytosis caused by rhamnolipid, produced by *P. aeruginosa* biofilms, in the chronically infected wounds, to ensure a fitness advantage and evasion of the immune system [28,29]. However, further insights on the actual impact of topical S100A8/A9 on PMN mobilization and physiology should be addressed in vitro.

The intervention did not have any significant impact on wound healing, as determined by planimetry, or on levels of EGF. This is probably due to the relatively short observation period (up to 10 days after wound infliction or six days post infection).

In conclusion, we have demonstrated that S100A8/A9 mitigates local *P. aeruginosa* biofilm infection by modulating local host response and attenuating systemic reaction in the chronic wound murine model. Accordingly, a clear growth inhibiting effect of recombinant murine S100A8/A9 on *P. aeruginosa* was observed in vitro. Apparently, this innate host defense protein possesses the capacity to combat an established biofilm infection as well as planktonic bacteria. The present study supports a beneficial role of S100A8/A9 as adjunctive treatment in refractory *P. aeruginosa* biofilm-infected wounds.

## 4. Materials and Methods

### 4.1. Study Design of Animal Experiments

Mice predisposed for chronic *P. aeruginosa* biofilm infection by infliction of burn wound full-thickness necrosis were treated daily with S100A8/A9 or vehicle (PBS) for a period of 5 days post infection. Wounds were assessed by quantitative bacteriology, quantification of key inflammatory cytokines and growth factors locally, and count of systemically polymorphonuclear (PMN) leukocytes. Digital photoplanimetry described development of wounds within the 5 day treatment period. Assessment of epidermal growth factor (EGF) was chosen as a marker for progression in wound closure.

### 4.2. Animals

In total, 78 female BALB/c mice (Taconic Europe A/S, Lille Skensved, Denmark) were used for the present study. For comparison of inflammatory reaction to the wound infliction and no consequent treatment, six sham control and burn wounded, but not infected, mice were included. Three mice were evaluated at 2 days and three mice at 4 days post wound infliction. An additional three burn wounded mice were sacrificed immediately after infection for presentation of baseline levels. Two untreated and unburned mice were kept for background levels. 

Animals were acclimatized for at least 1 week in the animal facilities before experimentation and were allowed free access to chow and water. Animal care was provided by trained personnel.

The study was approved by the Animal Ethics Committee of Denmark (2015-15-0201-00618, 15 July 2017) and all experiments were performed following National and European Union guidelines.

### 4.3. Establishment of Chronic Wound Infection

First, structural damage to the skin acting as a predisposing factor for settlement of infection was induced by establishment of third degree burn wounds [13,30]. The area of the thermal lesion was 1.7 × 1.7 cm (2.9 cm^2^) and mice were anesthetized by 0.3 mL hyp/midazolam.

Four days post wound infliction, 100 μL of a 10^7^/mL colony forming units (CFU). *P. aeruginosa* biofilm solution (10^6^ CFU/wound) was injected subcutaneously (s.c.), centrally, beneath the wound. The biofilm was prepared as previously described [31]. Treatment was initiated 24 h after biofilm injection to allow for the settlement of infection. Mice were sacrificed by intraperitoneal injection of a pentobarbiturate/lidocaine overdose after 1, 2, 3, or 5 days of treatment.

### 4.4. Immunomodulation of Murine Wounds

Recombinant mouse S100A8/A9 (Cloud Clone, Katy, TX, USA) was reconstituted in sterile-filtered PBS (0.22 μm) to reach a concentration of 4 μg/mL and 250 μL of the solution (1 μg) was given s.c. by injection centrally beneath the wounds. Controls received PBS from the same flask used for reconstitution. 

Treatment (+/− S100A8/A9) was given 1, 2, 3, 4, and 5 days after biofilm injection. Fourteen mice were treated for 1 day, 14 for 2 days, and 16 for 3 days. Eleven mice were given PBS for 5 days (one mouse died as a result of the burn wound infliction) and 12 mice were given S100A8/A9 for 5 days.

### 4.5. Collection of Wounds

To minimize tissue disruption, wounds were carefully retrieved (*n* = 76) with sterile scalpels. Each wound was placed in 2 mL sterile saline and kept on ice until homogenization for 20 s by 14,000 rpm using a Heidolph Silent Crusher M (Heidolph Instruments, Schwabach, Germany) followed by centrifugation for 15 min at 5000 rpm. Supernatants were sterile-filtered (0.22 μm) and kept at −80 °C until analysis.

### 4.6. Quantitative Bacteriology

Serial dilutions of wound homogenates were prepared in saline and aliquots of 0.1 mL were spread onto modified Conradi-Drigalski medium (State Serum Institute, Copenhagen, Denmark), selective for Gram-negative rods. CFUs were counted the following day. The actual numbers of CFUs in the samples were calculated by multiplication with the dilution factor.

### 4.7. Bacterial Growth Inhibition by S100A8/A9

The growth inhibitory effect of recombinant S100A8/A9 (Cloud-Clone Corps, Katy, TX, USA) on *P. aeruginosa* (PAO1, Iglewski) was assessed. Bacteria were grown from an overnight culture to stationary phase in a liquid culture of Luria-Bertani (LB) medium, and diluted ×100 before assays to an OD_600_ of 0.043. One hundred microliters (100 μL) of bacterial suspension was added to 100 μL of recombinant S100A8/A9 heterodimer to reach final concentrations of 40, 20, 10, 5, 2.5, 1.25, and 0 μg/mL and incubated with gentle shaking for 1–24 h at 37 °C. OD_600_ was measured in duplicates at 0, 2, 4, 6, and 24 h. Bacterial growth and inhibition was assessed by plating 10-fold serial dilutions of bacterial suspensions on agar plates at 1, 6, and 24 h, which were then incubated at 37 °C for 24 h. The actual growth was determined by count of CFU/mL. Data are expressed in log_10_ CFU/mL as a function of time and concentration.

### 4.8. Quantification of Inflammatory Cytokines and Growth Factors

Inflammatory markers were quantified in sterile-filtered wound homogenates. Levels of TNF-α, IFN-γ, IL-10, KC, IL-1β, G-CSF, and IL-17 (Bio-Rad, Hercules, CA, USA) were analyzed by Luminex Immunoassay (Luminex Corp., Austin, TX, USA). EGF levels were quantified by ELISA (Sigma Aldrich, Saint Louis, MO, USA), as S100A8/A9 also has stimulatory effects on normal, human keratinocytes [32].

### 4.9. Systemic Polymorphonuclear Count

Count of systemic PMNs in anticoagulated whole blood was performed immediately after sacrifice by flow cytometry, as described by Brochmann et al. [33]. In brief, distribution of PMNs and total white blood cells in anticoagulated blood from all mice was determined by use of a FACSCanto^TM^ flow cytometer (BD Biosciences, San Jose, CA, USA). A 488 nm argon laser and a 530/30 nm band pass emission filter were used for the recording of hydroxyphenyl fluorescence in FL-1. PI fluorescence was collected through a 585/42 nm band pass emission filter, recorded in FL-3. Samples were analyzed at a low flow rate (10 μL/min) and ≥10,000 events were recorded per sample. Cytometer Setup and Tracking Beads (BD Biosciences, San Jose, CA, USA) was used for calibration of the instrument. Flow data were processed and analyzed by Diva (BD Biosciences, San Jose, CA, USA).

### 4.10. Assessment of Wound Closure

All mice were photographed at a fixed distance by camera (CANON, EOS 650D, Kyushu, Japan) every other day. Mice were anesthetized by halothane during photography. Digital images were analyzed using photoplanimetry (Image J^®^, version 1.47, Bethesda, MD, USA). Necrotic area and total area of each wound were evaluated.

### 4.11. Statistics

Statistical calculations on continuous data were performed using the statistical programme GraphPad Prism (version 7.02; GraphPad Software, Inc., San Diego, CA, USA). Quantitative variables (bacteriology) were compared by student’s unpaired *t*-test. Cytokine assessment was by two-way ANOVA (Bonferroni). A level of *p* ≤ 0.05 was considered statistically significant.

## Supplementary Materials

Supplementary materials can be found at www.mdpi.com/1422-0067/18/7/1359/s1.
