# Supplementary material for: Immune Modulating Topical S100A8/A9 Inhibits Growth of Pseudomonas aeruginosa and Mitigates Biofilm Infection in Chronic Wounds
Source: Int J Mol Sci. 2017 Jun 26;18(7):1359. doi: 10.3390/ijms18071359 (PMC5535852; doi:10.3390/ijms18071359)
Supplement: Supplementary file 1 [file ijms-18-01359-s001.zip › ijms-196610 supplementary-done.docx]

Supplementary material

***Abbreviations:***

background (n=2),

2 DPB (days post burn) burned, two days after wounding, no infection

4 DPB: burned, 4 days after wounding; no infection

1 DPI (burned, infected): burn and infection, results obtained immediately after infection, no intervention by S100A8/A9 or PBS

D1: 1 day post infection, -: PBS, +: S100A8/A9

D5: 5 days post intervention.


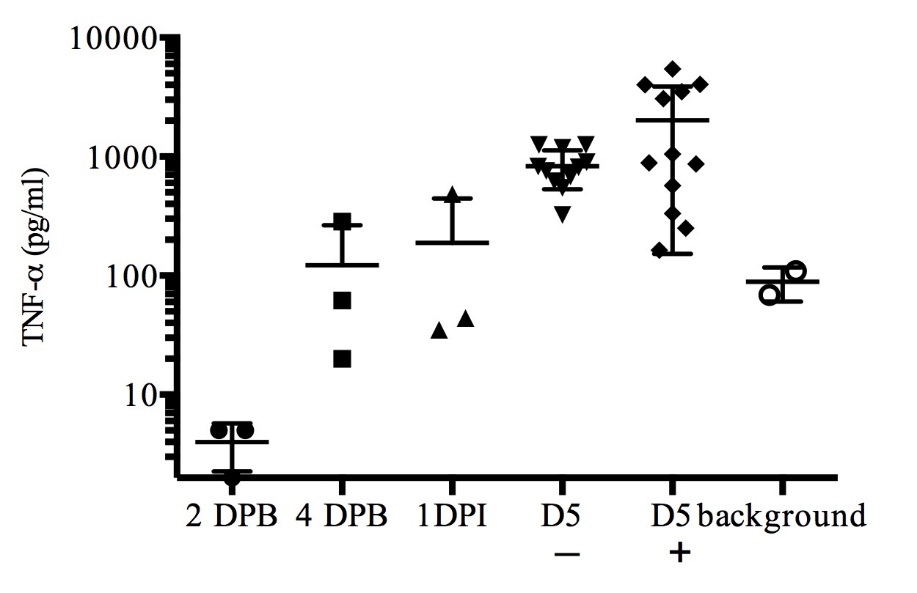


Figure S1. The impact of 5 days of treatment (PBS: − and S100A8/A9: +) on TNF-α levels

*
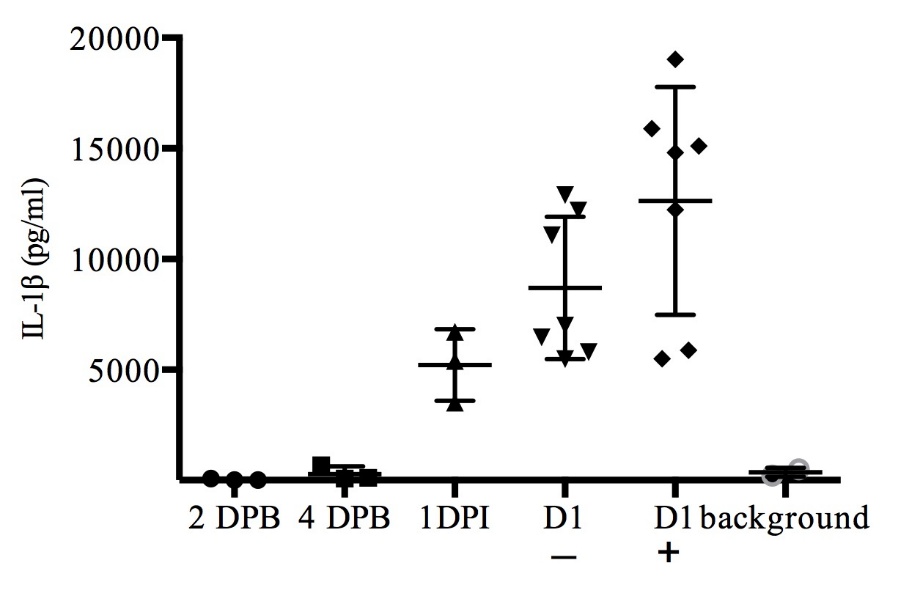
*

Figure S2. The impact of 1 day of treatment (PBS: - and S100A8/A9: +) on IL-1β levels.


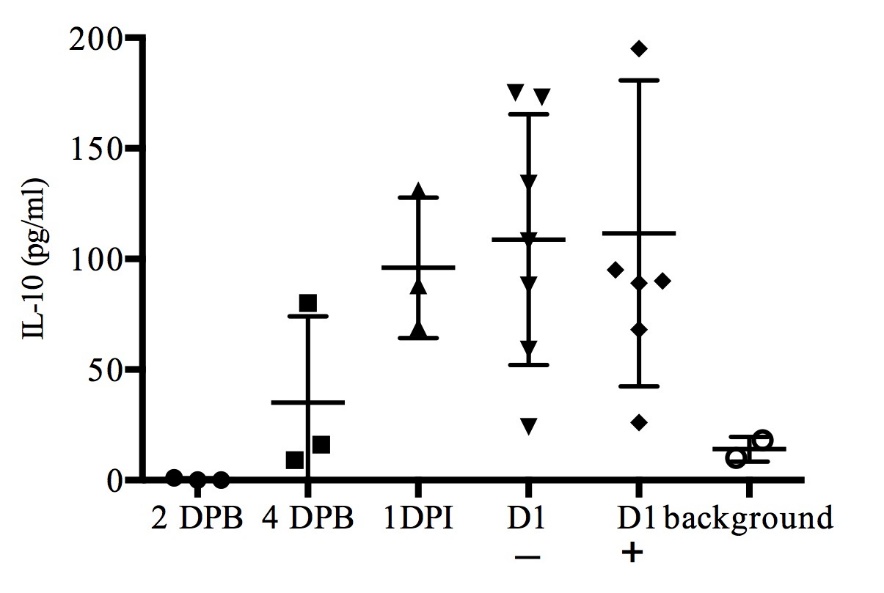


Figure S3. The impact of 1 day of treatment on IL-10 levels.


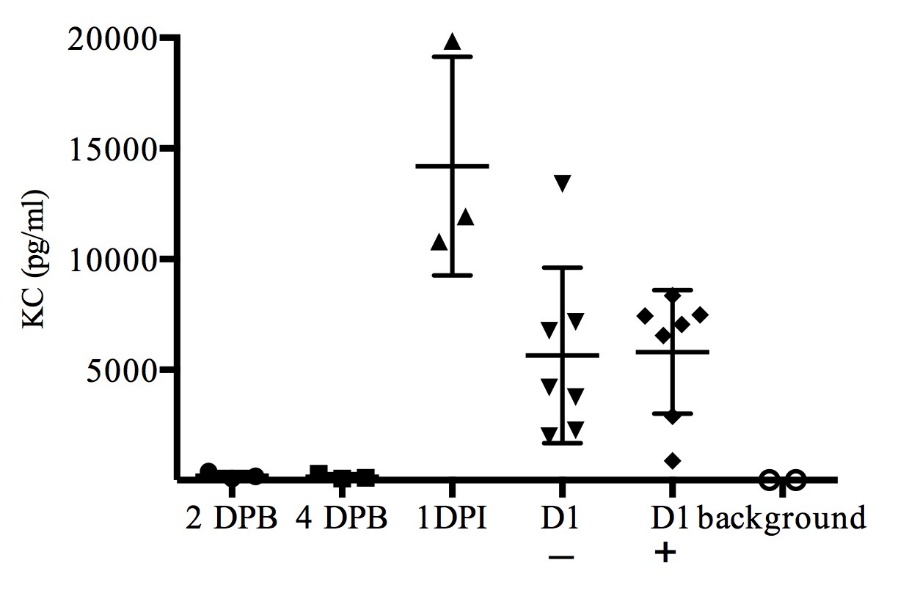


Figure S4. The impact of 1 day of treatment on KC levels.


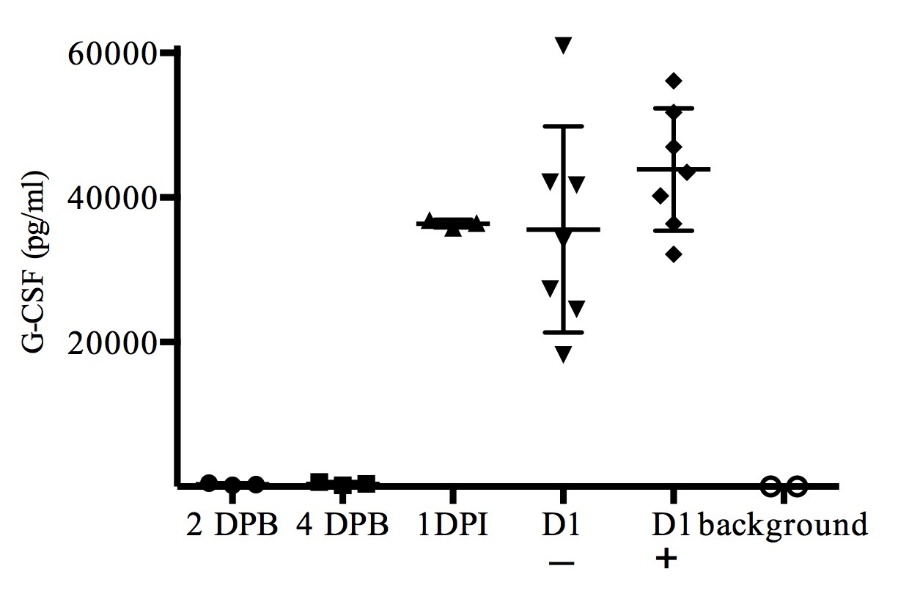


Figure S5. The impact of 1 day of treatment on G-CSF levels


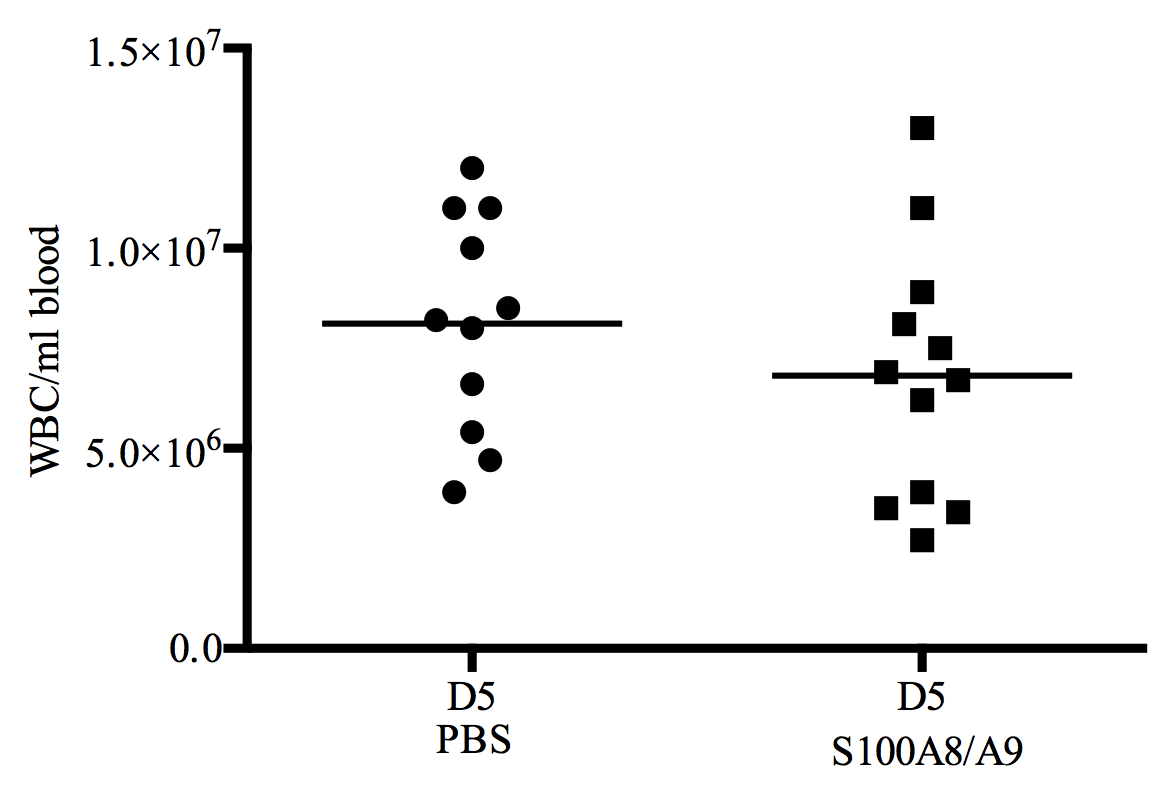


Figure S6. No significant impact on White Blood Cell (WBC) count was observed after 5 days of treatment.


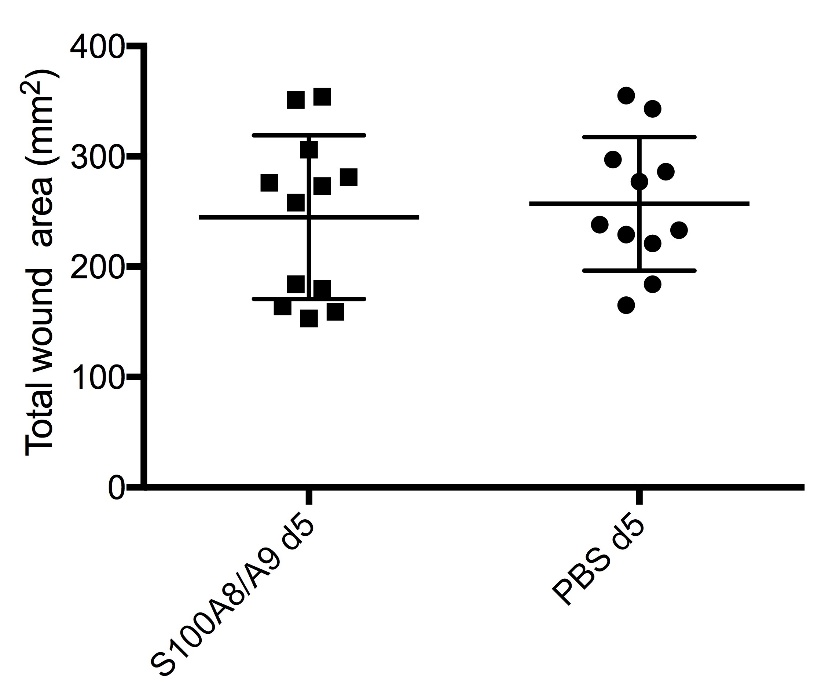


Figure S7. No significant changes in total wound area were observed after 5 days of treatment.


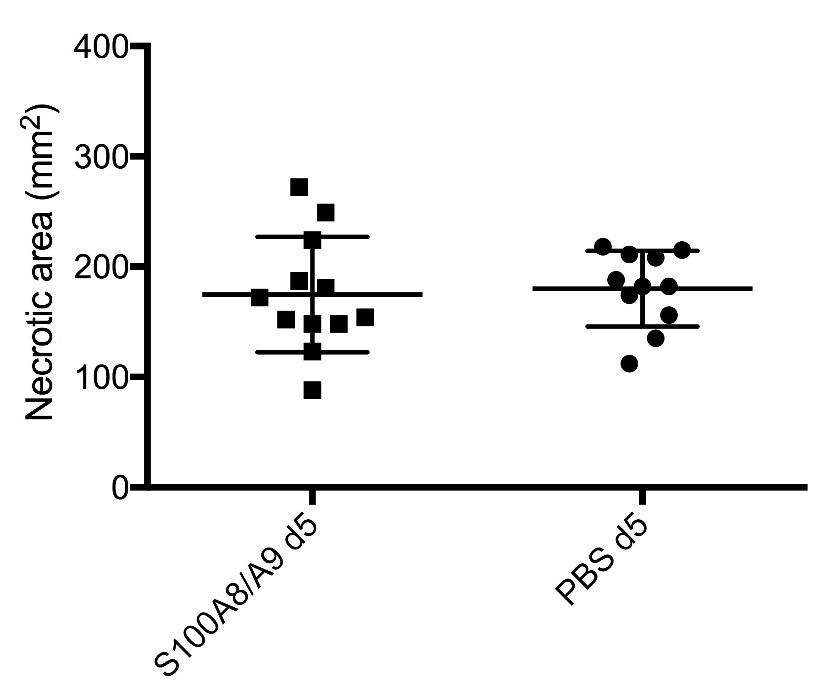


Figure S8. No significant differences in size of necrosis were observed after 5 days of treatment.

*
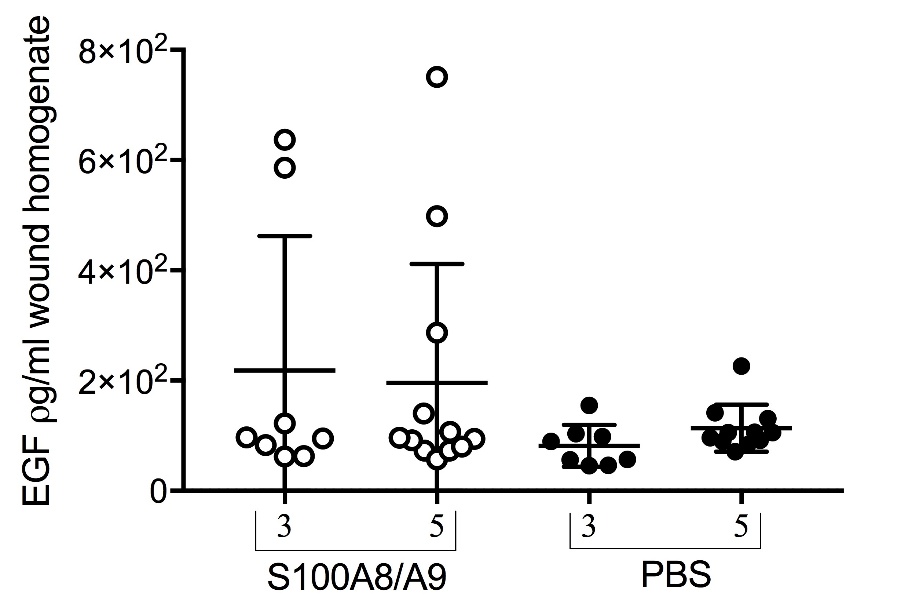
*

Figure S9. No significant differences in Epidermal Growth Factor levels were observed after 5 days of treatment.
